# What’s New? Gestures Accompany Inferable Rather Than Brand-New Referents in Discourse

**DOI:** 10.3389/fpsyg.2020.01935

**Published:** 2020-09-23

**Authors:** Sandra Debreslioska, Marianne Gullberg

**Affiliations:** ^1^Centre for Languages and Literature, Lund University, Lund, Sweden; ^2^Lund University Humanities Lab, Lund University, Lund, Sweden

**Keywords:** gestures, discourse, reference, information status, speech-gesture relationship

## Abstract

The literature on bimodal discourse reference has shown that gestures are sensitive to referents’ information status in discourse. Gestures occur more often with new referents/first mentions than with given referents/subsequent mentions. However, because not all new entities at first mention occur with gestures, the current study examines whether gestures are sensitive to a difference in information status between brand-new and inferable entities and variation in nominal definiteness. Unexpectedly, the results show that gestures are more frequent with inferable referents (hearer new but discourse old) than with brand-new referents (hearer new and discourse new). The findings reveal new aspects of the relationship between gestures and speech in discourse, specifically suggesting a complementary (disambiguating) function for gestures in the context of first mentioned discourse entities. The results thus highlight the multi-functionality of gestures in relation to speech.

## Introduction

When producing a stretch of discourse, speakers can use speech and speech-associated gestures to indicate to whom or what they are referring. Bimodal referring is a widely acknowledged phenomenon, but the mechanism explaining why gestures occur at specific moments when speakers mention entities in discourse is less well understood. McNeill (1992, 2005) proposes that communicative dynamism (CD) – the degree to which a piece of information “pushes the communication forward” (Firbas, 1971, p. 136) – determines the presence versus absence of gesture. McNeill takes information status, one of three factors influencing CD (Firbas, 1971), as a starting point and shows that the less accessible the information, the more likely a gesture is to occur. Conversely, the more accessible the information, the less likely a gesture is to occur. This would suggest that new entities in discourse are more likely to occur with gestures than already mentioned ones, an observation that is well supported in the literature (Marslen-Wilson et al., 1982; Levy and McNeill, 1992; McNeill and Levy, 1993; Gullberg, 1998, 2003, 2006; Levy and Fowler, 2000; Foraker, 2011).

However, there is evidence that not all entities which are mentioned for the first time in discourse, representing the lowest degree of accessibility (or highest degree of newness), are accompanied by gestures (e.g., Gullberg, 2003; Foraker, 2011). Hitherto, this variation has gone unmentioned. The current study therefore examines the variation in the incidence of gesture with entities mentioned for the first time and specifically probes the possibility that gesture production may be related to entities’ information status (brand-new vs. inferable; Prince, 1981; see also Clark, 1977; Fillmore, 1982; Chafe, 1994; Givón, 1995; Gundel, 1996), which in turn may interact with nominal definiteness [definite vs. indefinite noun phrases (NPs)].

### Speech-Associated Gestures

**Figure 1:**
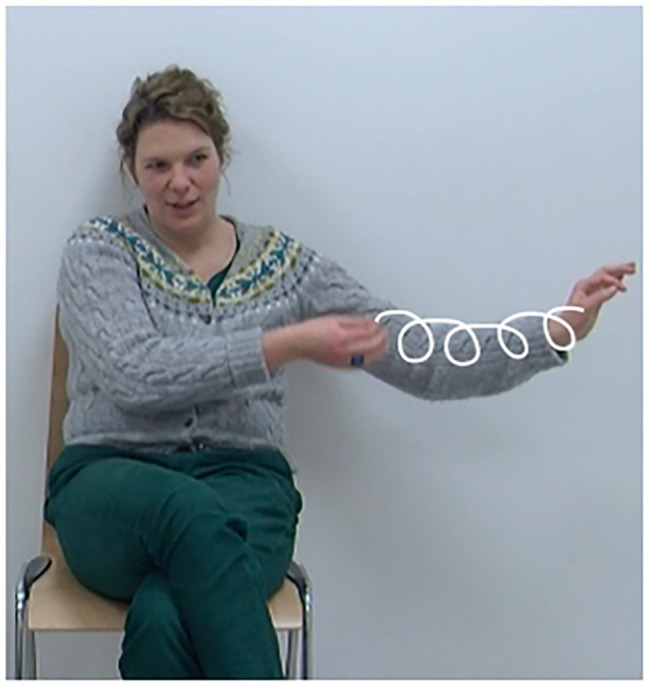
Iconic representation of “sewing a sleeve” (gesture alignment indicated in bold face). *Wie sie zuerst auf der Seite, auf der die Fee steht, **den Ärmel zun**äht* “How she **sews the sleeve** on the side, on which the fairy is standing”

**Figure 2:**
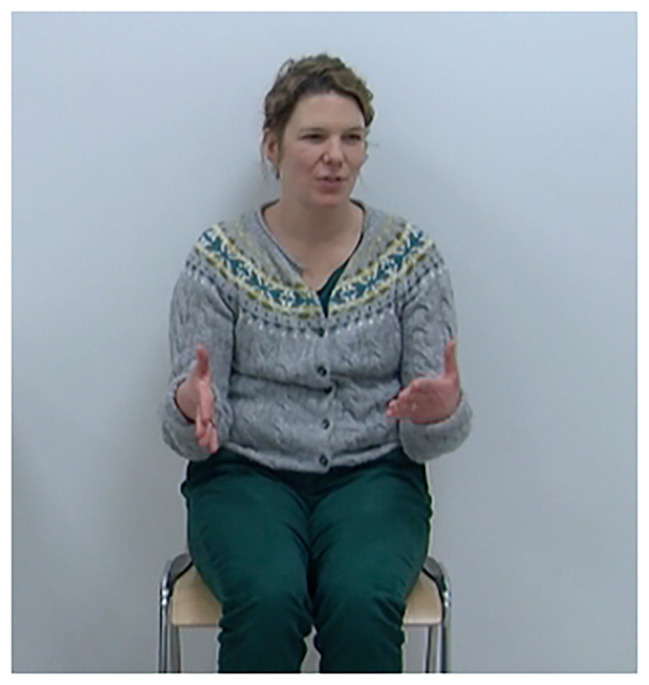
Iconic representation of “a table” (gesture alignment indicated in bold face). *Und es gibt **ein Tisch*** “And there is **a table**”

When speakers engage in talk, bodily action is always mobilized, which goes beyond the use of the anatomical apparatus needed for speaking (Kendon, 2014). This bodily action can involve the face and eyes, the neck and head, the upper body and trunk, and importantly, the hands and arms. A large body of research shows that the hand and arm movements speakers perform while speaking (also called gesticulations, co-speech gestures, speech-associated gestures, manual gestures, or simply gestures) are organized as patterns of movement that are rhythmically coordinated with speech production (Kendon, 1972, 1980). At the same time, they are also considered to be meaningful, specifically in how they relate to the meanings in the speech they accompany (McNeill, 1992; Kendon, 2004). For instance, speakers may use gestures to provide iconic representations of what is being talked about or they may use them to point to or locate entities (see Figures 1, 2). In Figure 1, the speaker mentions the entity *Ärmel* “sleeve” for the first time within a sewing event. In exact temporal co-occurrence with this mention, she uses a gesture to represent the sewing action performed on the sleeve by moving her right hand in a circular fashion along her left arm producing an iconic depiction. In Figure 2, the speaker mentions the existence of the entity *Tisch* “table” for the first time. She raises both hands in parallel from her lap to about chest level, with flat hands and palms facing each other, in order to indicate the shape/size of the table. This tight coordination in meaning and timing of two modalities is at the basis of the consideration that gestures and speech are conceptually linked (Kendon, 2004).

### Speech-Associated Gestures and the Information Status of Entities

The relationship between speech and gestures extends from the local level of one composite expression to more global interactions of the two modalities, as is the case for the organization of connected discourse. Gestures and speech vary in a coordinated fashion in the way they are deployed depending on the unfolding of information in discourse. For example, for the tracking of referents in discourse, a growing number of studies demonstrate a close link between gestures and speech, emphasizing the role played by the information status of entities. When entities are new or less accessible, they are typically expressed with richer referential expressions in speech (as in lexical NPs) and are accompanied by gestures. In contrast, when entities are given or more accessible, they are expressed with leaner referential expressions in speech (as in pronouns) and are typically not accompanied by gestures (e.g., Marslen-Wilson et al., 1982; Levy and McNeill, 1992; McNeill, 1992, 2005; McNeill and Levy, 1993; Gullberg, 1998, 2003, 2006; Levy and Fowler, 2000; Yoshioka, 2008; Wilkin and Holler, 2011; Parrill, 2012; Debreslioska et al., 2013; Perniss and Özyürek, 2015; Debreslioska and Gullberg, 2019; but see So et al., 2010 for different results when using a different gesture coding approach). This pattern reflects Givón’s so-called principle of quantity (Givón, 1983), which predicts more marking material for less accessible information and less marking material for more accessible information (see also Ariel, 1988, 1991, 1996; Prince, 1992; Gundel et al., 1993; Chafe, 1994; Arnold, 1998, 2008, 2010; and for child discourse, see e.g., Clancy, 1993; Hickmann and Hendriks, 1999; Allen and Schroder, 2003; Narasimhan et al., 2005; Serratrice, 2005; Allen, 2008). More importantly, the pattern is also at the heart of McNeill’s theory of CD and gestures, which posits that the more a piece of information “pushes the communication forward” (Firbas, 1971, p. 136), the more likely it is that a gesture co-occurs with it. The information status (or how accessible a referent is) is one important factor influencing the CD of an expression (Firbas, 1971). Findings showing the parallelism between speech and gesture to signal new information (richer referential expressions and gestures) versus given information (leaner referential expressions and few/no gestures) are considered to be support for McNeill’s theory.

An example of this pattern is illustrated in (1), taken from the data set of the current study. In order to signal that referents are new, indefinite lexical NPs are used in speech for the referents *Kerzen* “candles” in utterance 1, and *Fee* “fairy” in utterance 2. When the referent “candles” is mentioned for the second time in utterance 2, the speaker uses a pronoun to refer back to it (*die* “they”). In gesture, this alternation between richer/leaner expressions is reflected in a variation in gesture incidence. Both first mentions are accompanied by gestures (i.e., the referents “candles” and “fairy,” marked in bold face), but the subsequent mention of the referent “candles” by the pronoun *die* “they” is not.

(1)

1. Und auf der Torte ähm sind **Kerzen**_1_ drauf “and on the big cake are candles.”
2. Die_1_ werden angezündet von ähm **einer Fee**_2_ “they are being lit up by a fairy.”

Although the literature thus shows that new referents are more likely to occur with gestures than old/given ones, it also shows that not all first mentions are accompanied by gesture (e.g., 39.8% in Foraker, 2011; 75% in Gullberg, 2003). This observation, in turn, seems to challenge predictions derived from McNeill (1992, 2005). Since a referent mentioned for the first time should always push the communication forward (or carry higher CD), we might expect every first mention to be accompanied by gesture. But it is not. It remains unclear why this should be the case.

One possibility is that a more fine-grained notion of information status is needed to account for the incidence of gestures. Specifically, in the context of new information and first mentions, entities could be divided into those that are brand-new and those that are inferable from the preceding context. Prince (1981, 1992) defines brand-new entities as being new to the preceding discourse and also new to the addressee. Inferable entities, on the other hand, are new to the preceding discourse, but their existence can be inferred by the addressee. A referent is typically rendered inferable by virtue of a trigger entity, which has previously been mentioned in the discourse (Prince, 1981, 1992). For instance, inferable referents are entities that stand in a part/whole relationship or in a content/container relationship to already-mentioned entities. For example, if the referent *Besen* “broom” has already been mentioned in a particular stretch of discourse, then a current mention of the referent *Stiel* “broomstick” can be considered inferable. Similarly, if the referent *Salzstreuer* “saltshaker” has already been mentioned, then a current mention of *Salz* “salt” can be considered inferable information. Note that these kinds of relationships that give rise to inferables hold true even if in some cases a particular referent does not have a certain part or content (e.g., an empty saltshaker). It is considered sufficient that the relationship typically holds true (Birner, 2013). More recent accounts further argue that inferable information should rather be regarded as “hearer new” but “discourse old” (Birner and Ward, 1998; Birner, 2013). This view emerges from observations that inferable information is often used in sentence constructions which depend on “discourse old” information on the one hand and in constructions which depend on “hearer new” information on the other.

The variation in information status between brand-new versus inferable referents can be signaled in speech by a formal variation in nominal definiteness. Speakers are likely to refer to inferable entities with definite lexical NPs (also called bridging expressions, as in e.g., *the broomstick*) more often than to brand-new entities (e.g., *a broom*; Clark, 1975, 1977). In principle, however, inferable entities can be represented by both indefinite and definite lexical NPs (Prince, 1992; Gundel, 1996), as illustrated in examples (2–3), taken from the current data set. In each case, the speaker has already introduced a broom as a whole into the discourse. At a later point, one speaker mentions the referent “broomstick” by using an indefinite lexical NP (2), whereas the other speaker chooses a definite lexical NP (3). In order to avoid circularity (i.e., by assuming that each definite nominal used for a first mention automatically represents an inferable entity, and vice versa), we will keep the formal marking of nominal definiteness separate from information status while still assuming that the two measures will co-vary, such that inferables will be referred to with definites more often.

(2) PP1: *Der hat nen braunen Stiel und gelbe Borsten* “it has a brown broomstick and yellow bristles.”
(3) PP8: *Der Besenstiel ist braun* “the broomstick is brown.”

McNeill’s (1992, 2005) theory of CD and gesture, but also most other previous research on gestures in discourse, would predict that brand-new referents – which are “truly” new since they have never been mentioned and cannot be inferred from previously mentioned referents – should attract more gestures than inferable referents. Furthermore, if it is the case that indefinite lexical NPs signal brand-new referents more than definite lexical NPs, then they should also attract gestures more than definite lexical NPs (Debreslioska and Gullberg, 2019; but see Wilkin and Holler, 2011).

The current study seeks to test these predictions in order to further our understanding of when first mentions attract gestures or not.

### The Current Study

The current study examines when discourse entities that are mentioned *for the first time* co-occur with gestures and when they do not. Particularly, it explores two variables, information status (brand-new vs. inferable reference) and nominal definiteness (definite vs. indefinite nominals) to test whether these two factors are related to the incidence of gestures (presence vs. absence) in bimodal discourse.

For speech, we predict that (a) brand-new entities are more likely to be mentioned with indefinite nominals, and conversely, that inferable entities are more likely to be represented with definite nominals. For gesture, we predict that, if information status and definiteness have an effect on the incidence of gestures, (b) brand-new referents will co-occur with gestures more than inferable referents, and (c) indefinite lexical NPs will co-occur with gestures more than definite lexical NPs.

## Materials and Methods

### Participants

We invited 20 native German speakers (16 female, mean age = 26, range 20–39) to participate in the study at Ludwig-Maximilian University, Munich, Germany. All participants came with a native German-speaking friend who acted as listener. Everyone provided written consent.

### Materials/Design

**Figure 3:**
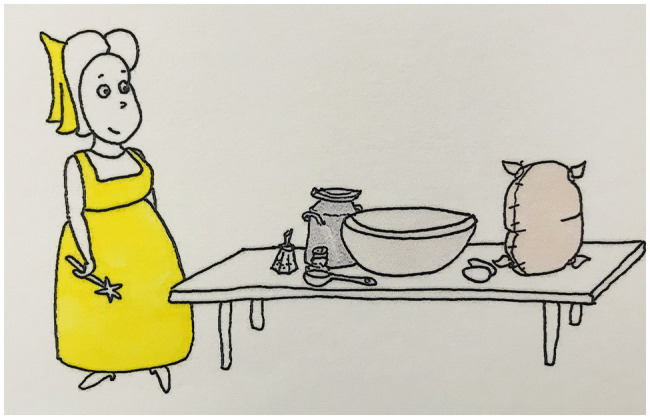
Example stimulus picture 1.

**Figure 4:**
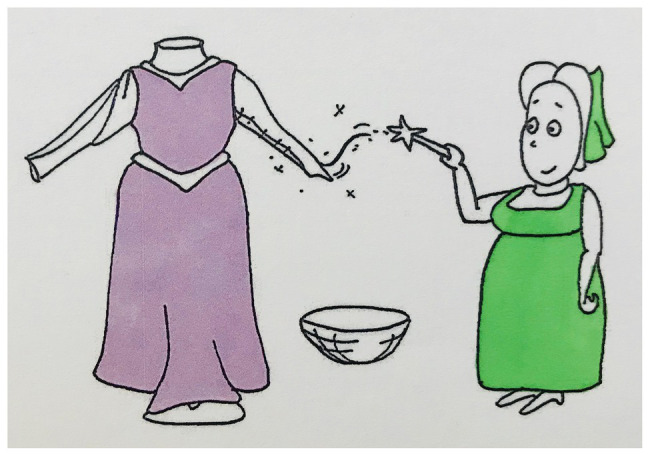
Example stimulus picture 2.

**Figure 5:**
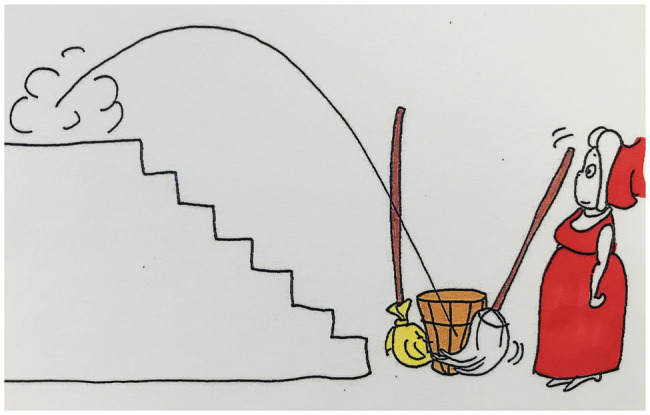
Example stimulus picture 3.

We used a picture story to elicit narrative speech and gestures. The story consisted of 127 pictures about three fairies, each having to fulfill a task (baking a cake, sewing a dress, and cleaning the floor), which they fail at, and consequently use magic to achieve (see Figures 3–5 for examples). References to the three fairies and a range of inanimate entities were considered (see Appendix B for a full list).

### Procedure

Participants sat across from each other and only the speaker was captured by a video camera, focusing on head and torso. Participants read instructions on paper, and the experimenter further repeated the main points orally to them. Speakers had to retell the picture story by answering the question “what happened?” Since the story was rather long, speakers only saw four to six pictures at a time, had unlimited time to memorize them, and then retold that piece to the listener before moving on to the next one. Speakers were encouraged to say something about each picture. The listener was not supposed to ask any questions, but to write down a short summary of each part of the story they just heard. While only the speaker was of interest for the current study, this was not disclosed to the participants. The listener was also instructed not to cross legs or arms in order to avoid mirroring by the speaker, which could be unfavorable for gesture production (e.g., Kendon, 1973; Chartrand and Bargh, 1999). The roles of speaker and listener were assigned randomly^1^. A session lasted between 45 and 90 min. The produced narratives were 20 min long on average. All participants were debriefed orally at the end of the experiment and were offered refreshments as compensation. Furthermore, all participants signed consent forms; while speakers also filled out a more detailed (language) background questionnaire based on work of Gullberg and Indefrey (2003).

### Speech Coding

A native speaker of German transcribed speech of all 20 narratives produced by the participants using German standard orthography, also taking note of filled pauses, word truncation, repetitions, etc. We then identified all referential expressions mentioning an entity for the first time. For the purposes of this study, we only selected references to concrete animate (i.e., the fairies) and inanimate entities (e.g., cake, broom, needle; see Appendix B for a full list of entities) that played a role in the story. We excluded all references to abstract/non-spatial/immaterial objects (as in 4). We also excluded references to “non-referential referents” (Chafe, 1994). Non-referential referents do not factually exist at the moment of mention, and speakers typically mention them in an irrealis context or present their existence as “hypothesized, predicted, or denied” (Chafe, 1994; example 5). Importantly, non-referential referents are not trackable and, thus, represent a different category of referents than those that are to be explored in the present study. Finally, we also excluded references to the pictures themselves (as in 6).

(4) *Sie hat eine Idee* “she has an idea.”
(5) *das soll vielleicht so ein Mehlsack sein* “it should perhaps be a bag of flour.”
(6) *Die grüne äh steht in der Mitte des Bildes* “the green fairy stands in the middle of the picture.”

Entities were either mentioned as core arguments (subjects and direct objects) in presentative utterances (such as existentials or locatives), transitive or intransitive clauses (corresponding to 60% of all referential expressions; see Table 1). In all three of these utterance types, the starting point is typically either an inanimate or animate locational element, the dummy subject *es* “it,” or the adverbial *da* “there,” and the first mentioned entities are placed toward the end of the utterance. In intransitive utterances, the speakers further use subject-verb inversion in order to place the first mentioned entity toward the end of the utterance. Placing the referents in utterance final (focal) position is typical in the context of first mentions. The rest of the entities were instantiated as either oblique arguments (29% of all referential expressions) or in verbless utterances (11% of all referential expressions; Table 1; for a construction type analysis and how different constructions are related to representational gestures, see Wu, 2018; Debreslioska and Gullberg, 2020).

**Table 1:** Clause types used to introduce referents and examples.

Clause types	Examples
Presentative clauses (existentials; locatives)	*und in dieser Schüssel sind drei Zauberstäbe* “and in the bowl are three wands” *es gibt einen Tisch* “there is a table” *da sind drei Feen* “there are three fairies” *die hat n Eimer* “she has a bucket”
Transitive clauses	*sie holt ein kleines Kästchen* “she goes to get a little box”
Intransitive clauses	*da kommen Funken raus* “there are coming out sparks” *dann fliegt ein Streichholz herbei* “then flies by a match”
Oblique arguments	*in einer Schüssel, hat sie die Kerzen* “in a bowl, she has the candles” *die eine läuft zum Tisch* “one of them walks to the table”
Verbless utterances	*und zwar mit roten Herzchen* “and namely/that is with red hearts”^1^ *und dann das Unterteil* “and then the lower part”^2^

1 Context of this verbless utterance: PP13: *Also die Tube mit dem Zuckerguss ähm verziert den Kuchen dann noch weiter und zwar mit roten Herzchen*. “So the icing bag continues to decorate the cake. And namely/that is with red hearts.”

2 Context of verbless utterance: PP22: *Aber die Nadel näht noch einmal das Oberteil besser zusammen und dann das Unterteil*. “But the needle sews together the upper part more appropriately. And then the lower part.”

#### Information Status

For each referential expression, we determined whether it referred to a brand-new or inferable entity. A brand-new entity was a “truly” new entity, which had never been mentioned before, and was not inferentially linked to a previous entity in the discourse. Conversely, an inferable entity corresponded to an entity that was mentioned for the first time, but that was linked to a previous “trigger” entity in the discourse *via* an inferential link (following Prince, 1981, 1992). In the current data set, two different links connected first mentions to previous entities, namely part/whole (e.g., sleeve – dress, egg shells – eggs), and content/container relationships (e.g., milk – milk can, sugar – sugar bowl; see Appendix B for a full list).

In relation to the way that entities were embedded in different utterance types, we observed that brand-new entities were introduced as core arguments in 67% of the cases, as oblique objects in 21% of the cases, and in verbless utterances in 12% of the cases. Inferable entities were mentioned as core arguments in 41% of the cases, as oblique objects in 50% of the cases, and in verbless utterances in 9% of the cases.

#### Noun Phrase Definiteness

We considered lexical NPs to be indefinite if they were mentioned as bare nouns, marked by indefinite determiners or numerals (*Milch/ein Besen* “milk/a broom”; *drei Feen* “three fairies”). We considered them to be definite when they were marked by definite determiners, such as definite articles, demonstrative pronouns and possessive pronouns (*die/diese Fee* “the/that fairy”; *ihr Kleid* “her dress”).

### Gesture Coding

We used frame-by-frame analysis of digital video in the software ELAN (Sloetjes and Wittenburg, 2008) to annotate manual gestures. We identified the most meaningful part of the gestural movements, the stroke phase (McNeill, 1992; Kendon, 2004), with sound turned off. We turn the sound off during the annotation of gesture phases to provide an objective and replicable annotation based on physical features of the hand/arm movements alone. We determined the onset and offset of a stroke when there were changes in the trajectory or movement of the hand(s), as well as when there were changes in the tension, shape, or placement of the hand(s) (see Kendon, 2004; Seyfeddinipur, 2006 for more detailed descriptions/instructions). In the case of deictic gestures, we counted the accelerated movement toward the end configuration together with the momentary stop in the end configuration as the stroke. For all other gestures, we also included post stroke holds, defined as movement cessations of the hand at the end of a gesture stroke, as meaningful parts of the gesture. One of the functions of post stroke holds is to allow for the rest of the co-expressive speech to be uttered before the hand goes into retraction or the next gesture (Kita, 1990; McNeill, 1992). They are therefore relevant for our analysis. Since the goal of the current examination is to find out when gestures are aligned with new referents in discourse, it is crucial to take into consideration the full chunk of speech that the meaningful part of the gesture is related to. In a last step, we identified which gestures co-occurred temporally with at least one syllable of the relevant referential expressions (following Stam, 2006; Gullberg et al., 2008) and only took those gestures into account for the analyses.

### Reliability Coding

A second German native speaker recoded speech for information status (brand-new vs. inferable) and nominal definiteness (indefinite vs. definite) for the referential expressions of four participants, corresponding to about 20% of the total amount of referential expressions used in the analyses. The agreement between coders was 90% for the coding of information status (brand-new vs. inferable). Interrater reliability was computed using Cohen’s kappa (Kappa =0.796, *SE* of kappa =0.035). The agreement between coders was 98% for nominal definiteness coding (indefinite vs. definite nominals). The interrater reliability was also measured using Cohen’s kappa (Kappa = 0.979, *SE* of kappa = 0.012).

A second coder recoded gestures for the same four participants in our data set, identifying gestures in the target utterances (i.e., those containing first mentions). The target gestures in those utterances constitute about 20% of the total amount of gestures that went into the analysis. Agreement was reached when the gesture that coder 2 identified aligned with the same referential expression as the one that coder 1 identified. The agreement between coders was 96%.

### Analyses

The analyses focus on first mentions of referents, brand-new or inferable, encoded by definite or indefinite nominals and produced with or without gestures. The data set consisted of 1,489 spoken referential expressions and 811 gestures produced by all 20 participants.

We used linear mixed effects models with the lmerTest package (Kuznetsova et al., 2017) in RStudio (RStudio Team, 2016) for all analyses. Table 2 summarizes the two main analyses. Analysis 1 concerns speech alone, examining the relationship between the information status of referents and their formal representation in speech as definite versus indefinite nominals. Analysis 2 then examines whether the presence of gesture is modulated by these variations in information status and definiteness.

**Table 2:** Variables and levels.

Analysis	Dependent variable	Levels	Predictor variable	Levels
1	Definiteness	Indefinite/Definite	Information status	Brand-New/Inferable
2	Presence of gesture	yes/no	Information status	Brand-New/Inferable
			Definiteness	Indefinite/Definite

## Results

### Speech

**Figure 6:**
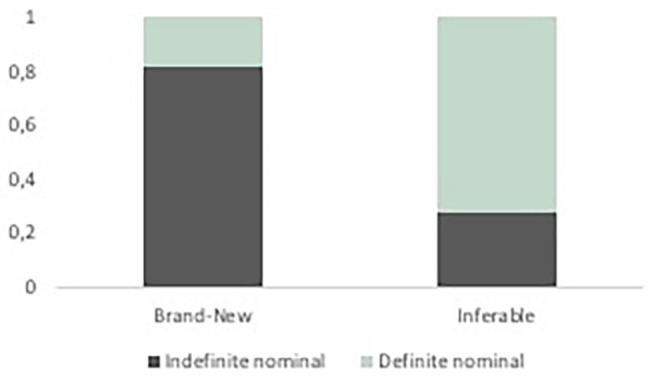
Indefinite nominals representing brand-new versus inferable entities (observed data).

In a first step, we explored the relationship between information status and definiteness in speech alone (Table 2, analysis 1). Figure 6 presents the observed distribution of indefinite nominals across brand-new (82%) and inferable referents (27%). The analyses revealed that, as expected, brand-new referents were significantly more likely to be expressed as indefinite nominal expressions than inferable referents (*EST* = −5.83, *SE* = 0.32, *z*-value = −18.46, *p* = 0.000). Conversely, inferable referents were significantly more likely to be encoded with definite than with indefinite nominal expressions (*EST* = 4.33, *SE* = 0.30, *z*-value = 14.43, *p* = 0.000).

### Gesture

**Figure 7:**
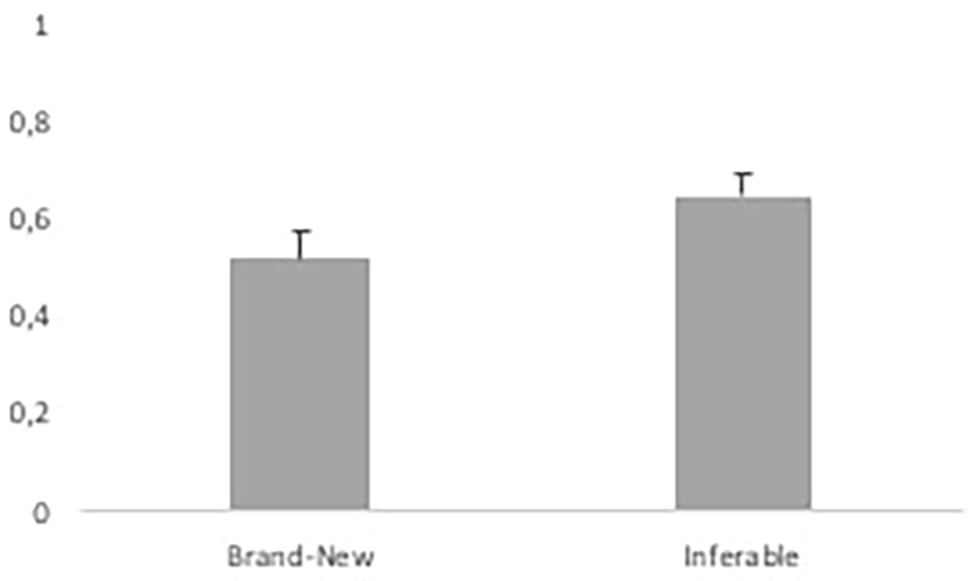
Mean proportions of gestures used with brand-new referents (0.52; *SE* = 0.05) versus inferable referents (0.65; *SE* = 0.5; observed data).

**Figure 8:**
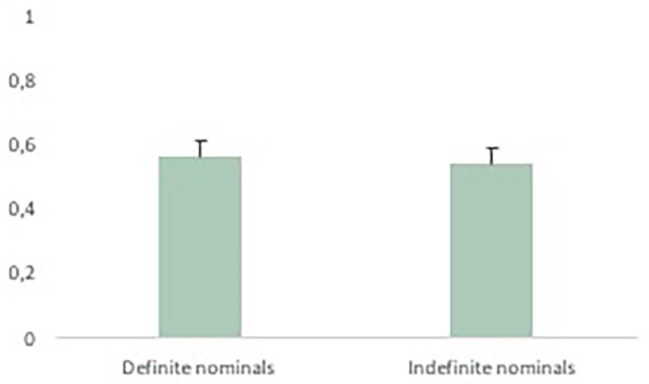
Mean proportions of gestures used with indefinite (0.54; *SE* = 0.5) versus definite nominals (0.56; *SE* = 0.05; observed data).

Next, we examined the relationship between the incidence of gestures and first mentions. We found that speakers produced gestures for 55% (*SD* = 23%) of all first mentioned entities (mirroring 60% in Foraker, 2011). We tested whether the incidence of gesture is modulated by two independent variables, namely information status operationalized as brand-new versus inferable, and referents’ representation in speech as indefinite versus definite nominals (Table 2, analysis 2). Figure 7 presents the observed distribution of (mean proportions of) gestures across inferable (65%) versus brand-new referents (52%). Figure 8 presents the observed distribution of (mean proportions of) gestures across definite (56%) versus indefinite (54%) nominals.

We ran five different models in order to determine the model that fit the data best. The first model included no predictor variables. The second and third models each included only one predictor variable, information status and definiteness, respectively. Finally, the fourth and fifth models included both predictor variables, but one was a simple model and the other an interaction model. All models included “subject” as a random predictor variable. We compared the Akaike information criterion (AIC) values between all models in order to determine the model which represented the best fit to the data set. Lower AIC values correspond to better fit (see Appendix A for a full list of models ranked according to their AIC values). More specifically, the AIC is an estimate of predictive accuracy, which measures how well a regression model will fit when applied to a new sample (see Long, 2012 for a detailed description).

The model comparisons showed that the simple model including the two predictor variables, information status and definiteness, best explained the present data. The analysis revealed that there was a significant effect of information status on the incidence of gestures but in the opposite direction from the prediction. Inferable referents were significantly more likely to occur with gestures than brand-new referents (*EST* = −0.73, *SE* = 0.16, *z*-value = −4.51, *p* < 0.000). There was no significant effect of definiteness (EST = −0.25, SE = 0.15, *z*-value = −1.68, *p* = 0.092).

## Discussion

The existing literature on discourse reference and gestures has shown that gestures are sensitive to referents’ information status in discourse such that they occur more often with new referents/first mentions than with given referents/subsequent mentions. However, because not all new entities are gestured about at their introduction, the current study set out to examine when first mentions of discourse entities are accompanied by gestures and when they are not. In particular, we considered the possible connection between gesture production and a more fine-grained difference in information status between brand-new and inferable entities, as well as the variation in linguistic encoding between indefinite and definite nominals, reflecting this difference in speech.

The results can be summarized in two points. First, the speech results showed that, as predicted, brand-new referents tend to be expressed by indefinite nominals (e.g., *a broom*), whereas inferable referents tend to be expressed by definite nominals (e.g., *the broomstick*). These findings are in line with previous research on this topic (e.g., Clark, 1975, 1977; Prince, 1981, 1992; Fraurud, 1990; see also Hickmann et al., 1996, for marking of newness in German), showing that referential form is sensitive to the inferability of referents mentioned for the first time.

**Figure 9:**
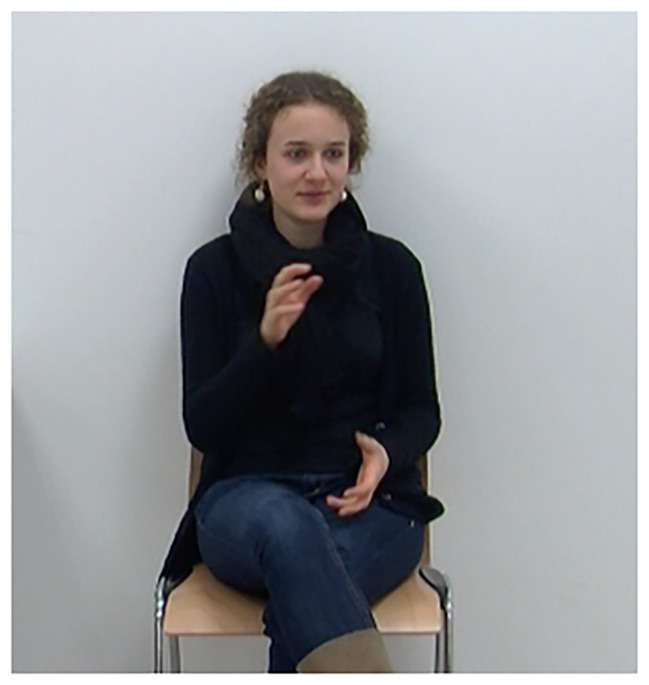
Example of a gesture accompanying the first mention of an inferable entity. *und man sieht jetzt das Ei**gelb*** “and one sees now the egg **yolk**”

Second, the gesture results revealed a link between gesture production and the brand-new/inferable distinction. Contrary to prediction, however, inferable referents were significantly more likely to be accompanied by gestures than brand-new ones. For example, the brand-new referent “dust pile” is introduced in a presentative utterance, *man sieht da vorne dran sonen kleinen Haufen* “one sees there in front a little pile,” and no gesture co-occurs with this first mention. Compare this to the first mention of the inferable referent “egg yolk” in the presentative utterance *und man sieht jetzt das Eigelb* “and one sees now the egg yolk,” in which a gesture localizing the egg yolk above a bowl accompanies the referential expression denoting it (Figure 9). In this example, the speaker raises her hand from her lap to about chest level while also using a marked hand shape to represent the shape of the egg yolk. Figure 9 illustrates the end position of her gesture.

This result poses a challenge to McNeill’s (1992, 2005) theory of CD and gestures, which posits that the more a piece of information pushes the communication forward, the more likely it is to co-occur with a gesture. It seemed plausible to assume that brand-new referents, which mark the lowest degree of accessibility of referents in discourse, push communication forward more than inferable referents and would thus be accompanied by gestures more often. However, the current results do not support this assumption.

The study asked whether information status plays a role for the incidence of gestures with first mentioned entities in discourse. The current results suggest that this is the case: gestures are significantly more likely to occur with inferable than with brand-new referents. Although these results go in unanticipated directions, they still suggest that gesture production is sensitive to the subtle distinction in information status suggested by the difference between brand-new and inferable referents. The findings therefore generally support previous research on the relationship between information status and gesture production in discourse (e.g., Marslen-Wilson et al., 1982; Levy and McNeill, 1992; McNeill and Levy, 1993; Levy and Fowler, 2000; Gullberg, 2003, 2006; Foraker, 2011). However, the question is why gestures should be more strongly linked to inferable than to brand-new entities. Birner (2013) proposes that inferable information is “discourse old” but “hearer new.” That is, inferable information can be considered “discourse old” because it is inferentially linked to the previous discourse in some way. But it is also “hearer new” because the information itself might not yet be active in the addressee’s representation of the discourse (even if in principle it might be more easily accessed than brand-new information). We suggest that speakers may use gestures to highlight these (inferable) pieces of information in order to signal to the addressee that, even if the information is marked by a definite determiner, they are still to add it as a new referent to the discourse representation. In other words, since inferable entities are linguistically encoded similarly to given information (by definite nominals), speakers may produce gestures more often with them to signal to the addressee that the information is not in fact given, but new since there is not yet any active representation of the information in the discourse model. By this account, gestures and speech in this particular context seem to work together in a complementary rather than a parallel fashion. That is, when speech does not provide an unambiguous cue as to whether information needs to be newly added to the discourse representation (such as by indefinite nominals), gestures can do so instead.

This interpretation is something of a departure from previous studies, which have mainly emphasized that the two modalities work in parallel. However, the interpretation is commensurate with McNeill’s (1992) view on gestures and speech as two dimensions of the same idea unit, where gestures do not always represent the same information as speech. The suggestion is that together, speech and gesture form a more complete representation. Similarly, Kendon (2014) suggests that gestures and speech together form a richer and more complex expression than if words or gestures are considered alone. In order to form such a complex expression, gestures can be used in flexible ways, as complements or supplements, sometimes even as substitutes or alternatives, to spoken expressions, always in accordance with the underlying communicative effort or intent (Kendon, 1986). The two modalities can thus be seen as adaptable resources allowing speakers to vary how they coordinate them depending on the communicative needs in different types of situations (Gullberg, 1998; Holler and Beattie, 2003; Kendon, 2004).

Interestingly, the results can also be related to qualitative observations in children’s speech and gesture production. Allen (2008) examined the influence of a referent’s information status on children’s argument realization in Inuktitut, a pro-drop language. She found that while children predominantly realize an argument overtly when it is “new,” there are still surprisingly many cases when children simply drop the argument even if the referent is new to the discourse. Qualitative analyses of some of the cases revealed that those elided arguments often refer to inferable referents instead of brand-new ones suggesting that children seem to differentiate between the two. More interestingly, Allen further showed that children tend to produce a gesture in place of the elided argument (while the timing of the gestures is unclear, we assume that gestures aligned with the verb phrases of an utterance; see also Yoshioka, 2005). That is, when referents represent new, but inferable information, children can drop the argument in speech and use a manual gesture instead. Often, this would be a deictic gesture pointing to the intended referent. Therefore, Allen’s (2008) analyses similarly suggest that when new but inferable information is linguistically treated like given information (i.e., by zero arguments in Allen’s study; by a definite determiner in the present study), a gesture might indicate the referent’s accessibility instead.

Importantly, although referent inferability explains a considerable part of the data, we still find inferable referents that are not accompanied by gestures (36%), as well as brand-new referents that do co-occur with gestures (52%). This means that there must be other aspects (possibly related to information status) which affect the presence of gestures in general, and with first mentions in particular. One aspect concerns the operationalization of inferability. In the current study we only considered inferential relations between first mentioned and already-mentioned trigger entities. Previous research, however, suggests that a first mentioned entity can also be inferentially related to a previously mentioned *activity*, *time*, or *place* (see e.g., Ward and Hirschberg, 1985; Ward and Prince, 1991; Ward and Birner, 2001). For instance, after having talked about a baking situation, a speaker might refer to the referent “spoon” with a definite nominal because she considers it inferable given that people often use spoons when baking. It is worth considering such relations in future studies.

A further aspect is more linguistic in nature. Firbas (1971), in his original work on CD in discourse, suggests that the amount of CD a speech unit carries (whether it is a referential expression, a verb or any other unit of meaning) does not solely depend on information status but also on the semantics and the word order used in a given utterance. It is therefore possible to complement an analysis of information status of first mentions with, for instance, the semantics of the verbs used to introduce an entity into discourse or the position of the referent in the utterance. It is already known that semantics plays an important role in the way that gestures represent information (e.g., McNeill, 1992, 2005; Kita and Özyürek, 2003; Kendon, 2004; Gullberg et al., 2008; Gullberg, 2009, 2011; Debreslioska and Gullberg, 2020). However, it is rather unclear whether and if so how the semantics of a referential expression and/or the verb used to introduce a referent would also affect the incidence of gestures. Other studies suggest a relationship between the way speakers package information morpho-syntactically and the way that gestures represent information (e.g., Kita and Özyürek, 2003; Özyürek et al., 2005; Kita et al., 2007; Gullberg et al., 2008). However, also for these studies, it is unclear how morpho-syntactic packaging would influence the incidence of gesture rather than the mode of representation in gesture. Thus, examining the interplay between semantics, word order, and information status in discourse might provide further useful insights into why some entities occur with gestures and others do not and on the relationship between gestures and speech on the discourse level more generally.

Finally, there are other non-discursive aspects to consider. For instance, some entity properties may be particularly conducive to gesture production. Different objects afford action on them to different degrees, which in turn may affect how likely people are to gesture about them. For example, Chu and Kita (2016) found that speakers produced speech-associated gestures more often when the stimulus objects they saw afforded action (i.e., objects with a smooth surface) than when they did not (i.e., objects with a spiky surface). Another issue is familiarity. For instance, if someone is not, or supposes the addressee is not, familiar with a certain entity or action, such as decorating a cake with an icing bag, they might be more likely to gesture about it (cf. Campisi and Özyürek, 2013). Lastly, of course, it is also possible that the specific task in this study might have influenced why speakers did or did not gesture about entities. For instance, we encouraged speakers to say something about each picture, which might have led them to talk about aspects of the stories that they would have left out otherwise. When speakers leave out information in a narrative context, it is typically because the information is not relevant to the story at hand or because the information is old/given. It is therefore possible that this is the reason why some speakers refrained from gesturing about certain entities they talked about. These suggestions will have to be explored in future studies. In particular, it would be desirable to design experiments which can tease apart the different levels that seem to influence the distribution of gestures (discursive and non-discursive).

In conclusion, the study has provided new evidence that the incidence of gestures in discourse is related to the referential status of entities. The focus on *first mentions* in relation to gesture is novel and, unlike previous studies on this topic suggesting a parallel link between the modalities, this study reveals a complementary function of speech and gestures in discourse. Specifically, gestures are shown to accompany first mentioned inferable referents, which are hearer new, but discourse old, more often than first mentioned brand-new referents, which are hearer new *and* discourse new. We propose that speakers use gestures to signal that inferable referents, despite their inferential link to the previous discourse, are hearer new and that, consequently, addressees need to add them as new to their discourse representation. Gestures may help them do this. The findings are in line with the view that gestures and speech work together to build a coherent piece of discourse, but they further highlight the many and flexible functions that gestures can fulfill in relation to speech in general and in bimodal discourse reference in particular.

## Data Availability Statement

The raw data supporting the conclusions of this article will be made available by the authors, without undue reservation.

## Ethics Statement

Ethical review and approval was not required for the study on human participants in accordance with the local legislation and institutional requirements. The patients/participants provided their written informed consent to participate in this study. Written informed consent was obtained from the individual(s) for the publication of any potentially identifiable images or data included in this article.

## Author Contributions

Both authors were involved in the conception of the work and the interpretation of the data. SD wrote the first draft of the paper and both authors were involved in the critical revision of the paper. Both authors approve the publication of the paper and agree to be accountable for all aspects of the work.

### Conflict of Interest

The authors declare that the research was conducted in the absence of any commercial or financial relationships that could be construed as a potential conflict of interest.

## Footnotes

^1^However, if one of the participants knew that the experimenter researched gestures (e.g., if a research assistant from the local university working on the topic of gestures came with a friend), then she was automatically assigned as listener.

## Supplementary Material

The dataset generated for this study can be found online at: https://www.frontiersin.org/articles/10.3389/fpsyg.2020.01935/full#supplementary-material
