# Supplementary material for: What’s New? Gestures Accompany Inferable Rather Than Brand-New Referents in Discourse
Source: Front Psychol. 2020 Sep 23;11:1935. doi: 10.3389/fpsyg.2020.01935 (PMC7539624; doi:10.3389/fpsyg.2020.01935)
Supplement: Supplementary file 1 [file Data_Sheet_1.docx]

## Appendices

### Appendix A: Model selection on the basis of AIC (Long, 2012)

|  | Specification | Predictors | AIC | Rank |
| --- | --- | --- | --- | --- |
| Model 1 | Gesture ~ 1 + (1\|Subject) | none | 1795.1 | 4 |
| Model 2 | Gesture ~ information status + (1\|Subject) | 1 | 1778.4 | 2 |
| Model 3 | Gesture ~ definiteness + (1\|Subject) | 1 | 1796.4 | 5 |
| Model 4 | Gesture ~ information status + definiteness + (1\|Subject) | 2 (simple) | 1777.5 | 1 |
| Model 5 | Gesture ~ information status * definiteness + (1\|Subject) | 2 (interaction) | 1779.4 | 3 |

### Appendix B: List of entities

Fairy 1, Fairy 2, Fairy 3, Broom, Bucket, Mop, Pile of dust, Wand(s), Hat, Fairy’s dress, Leaves, Stairs, Stars/sparks, Pot, Bowl, Table, Cake, Candles, Flame, Cleaning agent, Water, Match, Cook book, Milk can , Saltshaker, Sugar bowl, Bowl, Spoon, Flour bag, Eggs, Icing bad, Dough, Sugar hearts, Sugar dots, Sprinkles, Shoes, Mannequin, Dress, Needle(s), Thread, Basket, Belt, Scissors, Pieces of cloth (different colors), Collar, Box, Cloth triangle, Bow(s)

#### Parts/content of entities:

Broomstick, Bristle, Border of bucket, Hand, Arm, Head, Eye, Foot, Buttocks, Face, Shoulders, Finger, Mouth, Parts of the dress (upper, lower, skirt part, edge, neckline, sleeves), Parts of mannequin (hip, upper/lower body, arms, waist, shoulder, belly), Parts of cloth, Parts of stairs (stair head, steps), Corner/part of book, Egg yolk, Egg white, Egg shells, Salt, Sugar, Flour, Milk, Parts of table (egde, middle)
